# Modeling of adsorption of Methylene Blue dye on Ho-CaWO_4_ nanoparticles using Response Surface Methodology (RSM) and Artificial Neural Network (ANN) techniques

**DOI:** 10.1016/j.mex.2019.07.016

**Published:** 2019-07-19

**Authors:** Chinenye Adaobi Igwegbe, Leili Mohmmadi, Shahin Ahmadi, Abbas Rahdar, Danial Khadkhodaiy, Rahmin Dehghani, Somayeh Rahdar

**Affiliations:** a Department of Chemical Engineering, Nnamdi Azikiwe University, Awka, Nigeria; b Department of Environmental Health, Zabol University of Medical Sciences, Zahedan, Iran; c Department of Environmental Health, Zabol University of Medical Sciences, Zabol, Iran; d Department of Physics, University of Zabol, Zabol, P.O. Box. 35856-98613, Iran; e Department of Environmental Health, Karman University of Medical Sciences, Karman, Iran

**Keywords:** Methylene Blue, Nanoparticles, Artificial Neural Network, Adsorption, Central composite design, Response Surface Methodology

## Abstract

The aim of this study is to evaluate the applicability of Ho-CaWO_4_ nanoparticles prepared using the hydrothermal method for the removal of Methylene Blue (MB) from aqueous solution using adsorption process. The effects of contact time, Ho-CaWO_4_ nanoparticles dose and initial MB concentration on the removal of MB were studied using the central composite design (CCD) method. Response Surface Methodology (RSM) and Artificial Neural Network (ANN) modeling techniques were applied to model the process and their performance and predictive capabilities of the response (removal efficiency) was also examined. The adsorption process was optimized using the RSM and the optimum conditions were determined. The process was also modelled using the adsorption isotherm and kinetic models. The ANN and RSM model showed adequate prediction of the response, with absolute average deviation (AAD) of 0.001 and 0.320 and root mean squared error (RMSE) of 0.119 and 0.993, respectively. The RSM model was found to be more acceptable since it has the lowest RMSE and AAD compared to the ANN model. Optimum MB removal of 71.17% was obtained at pH of 2.03, contact time of 15.16 min, Ho-CaWO_4_ nanoparticles dose of 1.91 g/L, and MB concentration of 100.65 mg/L. Maximum adsorption capacity (*q_m_*) of 103.09 mg/g was obtained. The experimental data of MB adsorption on Ho-CaWO_4_ nanoparticles followed the Freundlich isotherm and pseudo-second-order kinetic models than the other models. It could be concluded that the prepared Ho-CaWO_4_ nanoparticles can be used efficiently for the removal of MB and also, the process can be optimized to maximize the removal of MB.

• Synthesis and characterization of Ho-CaWO_4_ nanoparticles.
• Modelling and optimization of Methylene Blue removal onto Ho-CaWO_4_ using Response Surface Methodology (RSM) and Artificial neural network (ANN).
• Evaluation of the isotherm and kinetic parameters of the adsorption process.

## Graphical abstract

**Figure:**
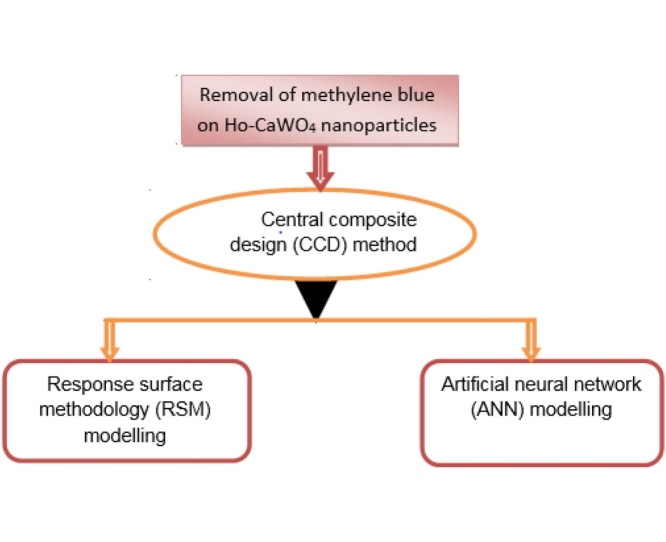

**Specifications Table**

**Table:** 

Subject Area:	Environmental Engineering
More specific subject area:	Adsorption
Protocol name:	Modeling of adsorption of Methylene Blue Dye on Ho-CaWO_4_ nanoparticles using Response Surface Methodology (RSM) and Artificial Neural Network (ANN) techniques
Type of data:	Image, table, and figure
How data was acquired:	All adsorption experiments were done in batch mode using the central composite design (CCD) method. After the adsorption process, the residual Methylene Blue (MB) concentrations were estimated. The initial and residual MB concentrations in the solutions were analyzed using a UV–vis recording spectrophotometer (Shimadzu Model, CE-1021-UK) at *λ*_max_ of 668 nm. Fourier-transform infrared spectroscopy (FT-IR) was done on a JASCO 640 plus to determine the functional groups present in the adsorbent (Ho-CaWO_4_ nanoparticles) before and after MB adsorption. Scanning electron microscopy (SEM) image of the adsorbent was obtained using an LEO instrument. The pH of the solution was measured using a MIT65 pH meter. The RSM and ANN data were collected via the Design Expert software (Stat-Ease, 8.0.7.1 trial version) and MATLAB (The Math Works Inc. 2018a), respectively.
Data format:	Raw and analyzed
Experimental factors:	The influence of pH, contact time, initial MB concentration and Ho-CaWO_4_ nanoparticles dose on the adsorption process. Kinetic and isotherm parameters were also presented.
Trial registration:	Not applicable
Name and reference of original method:	S. Ahmadi, L. Mohammadi, C A. Igwegbe, S. Rahdar, A. Banach, Application of Response Surface Methodology in the degradation of Reactive Blue 19 using H_2_O_2_/MgO nanoparticles advanced oxidation process, International J. Industrial Chem. 9 (2018) 241–253 (Published) [35].
Resource availability:	N/A

**Value of the Protocol**

**Table:** 

• The presented data established that Ho-CaWO4 nanoparticles can be applied for the removal of MB with great efficiency. • Data on the adsorption isotherm, kinetics, Response surface methodology (RSM), Artificial neural network (ANN) and effect of process variables were provided, which can be further explored for the design of a treatment plant for the treatment of MB containing industrial effluents where a continuous removal is needed on a large scale. • FTIR and FE-SEM data for Ho-CaWO4 nanoparticles were also provided. • The dataset will also serve as reference material to any researcher in this field.

## Description of protocol

Recently, the increasing number of emerging contaminants of high concern resulting from industrial and human-made activities present problems to the environment [[1], [2], [3]]. The textile industry is one of the most important industries around the world which demands large volumes of water in different areas, and also the source of colored and toxic wastewaters [4]. Industrial dyes or colors are amongst the top priority environmental pollutants found in industrial wastewaters [3] which are imperative due to several reasons including reduction of light permeability which may, in turn, result in impaired photosynthesis in water resources [5]. Methylene Blue (MB) is a cationic dye with a complex aromatic structure which is used for coloring cotton and silk [6]. This compound can cause impaired respiration. Furthermore, direct exposure to these dyes causes permanent damage to the human and animal eyes; they can also lead to local burns, nausea and vomiting, mental disorders, and Methemoglobinemia [6,7].

Several treatment methods have been proposed for the removal of dyes from contaminated waters which include photodecomposition, electrolysis, adsorption, oxidation, biodegradation and coagulation–flocculation [[7], [8], [9], [10], [11], [12]]. Amongst the different physical and chemical treatment processes, adsorption is an effective technique which is successfully used for the removal of colors from wastewaters [7].

Among the different adsorbents, nanoparticles have been revealed to possess great potential for the adsorption of organic compounds especially colors from wastewaters and sewage tanks due to their high surface to volume ratio [13,14]. Therefore, research on nanotechnology and its development have increased immensely [15]. In the biological synthesis of nanoparticles, harmful chemical compounds and solvents which are used in chemical methods of synthesis are replaced with natural compounds and biological agents in plant extracts such as enzymes, carbohydrates, and terpenoids [16]. Thus, the synthesis of nanoparticles using natural resources leads to reduced stages of synthesis and less usage of environmentally degrading energy and chemical solvents. The use of environmentally friendly materials such as starch and maltose in this study is a green approach [17]. Nanoparticles of Ho-CaWO_4_ have been synthesized through diverse methods such as chemical precipitation [18], microwave radiation [19], hydrothermal [20] and sol-gel [21] methods.

Optimization studies have been done effectively using the Response Surface Methodology (RSM) statistical technique [22]. Response Surface Methodology (RSM) has been broadly applied for the improvement of products and processes [23]. The RSM reduces the number of experimental runs and the time required to carry out a series of experiments [24]. In recent times, artificial neural works (ANN) are used for the prediction of responses in different disciplines due to their ability to employ learning algorithms and distinguish the relationships between the input and output for nonlinear systems [[22], [23], [24], [25], [26], [27]]. The comparison of the predictive and capabilities of the RSM and ANN modeling techniques have been studied by different authors [[22], [23], [24], [25], [26], [27], [28], [29], [30]]. All authors mentioned above proved that the ANN has an edge over RSM in predicting responses of systems except for Ghosh et al. [22] who disproved the notion.

The purpose of this study is to optimize the adsorptive removal of Methylene Blue dye on Ho-CaWO_4_ nanoparticles using the Response Surface Methodology based on the Central Composite Design (CCD). The performance and capability of the RSM and ANN for predicting the output responses were also compared. The CCD was used because it gives a higher prediction of the response [22]. The RSM was also applied to determine the optimum conditions of the process variables including pH, time, Ho-CaWO_4_ nanoparticles dose and initial MB concentration, and a predictive model equation for the adsorption process was also generated. The isotherm and kinetics of the process were also studied.

## Material and methods

### Chemicals and apparatus

Methylene Blue (MB) with a molar mass of 319.85 g/mol, molecular formula of C_16_H_18_N_3_CLS, p*K*_a_ of 3.5 and wavelength of maximum absorption (*λ*_max_) of 668 nm waspurchased from Alvan Hamedan, Iran.

### Synthesis of Ho-CaWO_4_ nanoparticles (Ho-CaWO_4_NPs)

Sucrose was used as a masking agent to wash the nanoparticles of Ho-CaWO_4_ using the hydrothermal method. 0.2 mol of calcium salt ($\text{C}\text{a}{\left({\text{N}\text{O}}_{3}\right)}_{2}.6{\text{H}}_{2}$O) was dissolved in 20 ml of distilled water in a beaker and then, 0.2 mol of sucrose solution was added. After vigorous stirring for 30 min, Holmium salt ($\text{H}\text{o}{\left({\text{N}\text{O}}_{3}\right)}_{3}.6{\text{H}}_{2}$O) was added to the reaction container in the ratio of 2%. The resulting solution was dissolved in 10 ml distilled water, and then 0.2 mol of ${\text{N}\text{a}}_{2}{\text{W}\text{o}}_{4}.2{\text{H}}_{2}$O was added and allowed to stand. After 1 h, the sample was kept in an autoclave for 18 hat 160 ℃. The autoclaved sample was washed with distilled water and ethanol, and dried in an oven at 70 ℃. The resultant solution was calcified for 4 hat 700 ℃  in a furnace.

### Characterization of the synthesized Ho-CaWO_4_ nanoparticles

Fourier transform infrared spectroscopy (FT-IR) was applied to dictate the functional groups participating in the adsorptive degradation of MB. The FT-IR spectra of the Ho-CaWO_4_ nanoparticles were acquired using a Nicolet Magna 550 spectrometer in KBr with a scan range of 400–4000 cm^−1^. Scanning electron microscopy (SEM) was used to examine the morphological structure of the Ho-CaWO_4_ nanoparticles using an LEO instrument.

### Batch experiments

The effects of Ho-CaWO_4_ nanoparticles dose (0.1–0.4 g/L), contact time (30–120 min), pH (3–11) and initial MB concentrations (20–80 mg/L) on MB removal were investigated. To work in a discontinuous system, Erlenmeyer flasks of 250 ml were used. For each adsorption experiment, 100 ml of MB solution with a specified initial concentration was added into the Erlenmeyer flasks. The desired pH was set. The pH of the solution was adjusted using 0.1 N HCl or 0.1 N NaOH solutions. A known dose of adsorbent was added to the flasks and then mixed in a magnetic stirrer at 180 rpm for 2 h. The residual MB concentrations were measured usinga UV–vis spectrophotometer (Shimadzu Model: CE-1021) at *λ*_max_ of 668 nm. The amount of MB adsorbed on the Ho-CaWO_4_ nanoparticles, *q_e_*was obtained as follows [31,32]:

(1) $$q_{e}=\frac{(C_{0}-C_{e})V}{M}$$

Also, the removal efficiency, %*R* was calculated based on the following formula [33]:

(2) $$\%R=\frac{\left(C_{0}-C_{f}\right)}{C_{0}}100$$

Where *C_0_* is the initial MB concentration, *_Ce_* is the equilibrium liquid phase concentration of MB (mg/L), *C_f_* is the final concentration, *V* is the volume of the solution (L) and *M* is the amount of adsorbent used(g).

### Design of experiments and statistical analysis

Central composite design (CCD) was used to design the experiments for the adsorption of MB on Ho-CaWO_4_ nanoparticles using the Design Expert software (Stat-Ease, 8.0.7.1 trial version). Four factors (the independent variables) including initial pH, contact time, Ho-CaWO_4_ nano dose and initial MB concentration at three levels of small factorial face-centered CCD based on RSM (Table 1) was used which gave a total of 21 experimental runs (Table 2). The operating variables were coded according to Eq. (3) [34]:

(3) $$Xi=\frac{\left(X_{i}-X_{0}\right)}{\Delta X}\times 100$$

Where *X_i_* is the coded value of the independent variable, *X*_0_ is the value of *X_i_* at the center point and Δ*X* is the step change value.

**Table 1:** The experimental range and levels of independent process variables assessed.

Independent Variables	Notation	Unit	Range and levels of actual and coded values		
			−1	0	+1
initial pH	A (X_1_)		2	6	10
Time	B (X_2_)	min	15	62.5	110
Dosage	C (X_3_)	g/L	1	0.01	2
Concentration	D (X_4_)	mg/L	100	250	400

**Table 2:** Experimental design matrix with the experimental and predicted values for MB adsorption on Ho-CaWO_4_ nanoparticles using the RSM and ANN modelling techniques.

Run	pH	Time (min)	Nano dose (g/L)	Concentration (mg/L)	Removal efficiency (%)		
					Actual	Predicted	
						RSM	ANN
1	10	110	2	100	60.33	60.39	60.33
2	10	110	0.01	100	60.92	60.84	60.92
3	10	15	2	400	50.50	50.56	50.50
4	2	110	0.01	400	56.25	56.18	56.07
5	10	15	0.01	400	50.04	49.97	50.04
6	2	15	2	100	71.17	71.22	71.17
7	2	110	2	400	56.96	57.02	56.96
8	2	15	0.01	100	71.00	70.93	69.24
9	2	62.5	1	250	63.17	63.21	65.11
10	10	62.5	1	250	55.67	55.71	55.67
11	6	15	1	250	58.67	58.71	58.67
12	6	110	1	250	56.09	56.12	56.09
13	6	62.5	0.01	250	58.33	58.63	62.03
14	6	62.5	2	250	59.17	58.95	59.17
15	6	62.5	1	100	67.00	67.04	67.00
16	6	62.5	1	400	55.00	55.04	55.00
17	6	62.5	1	250	59.17	59.02	59.09
18	6	62.5	1	250	59.30	59.02	59.09
19	6	62.5	1	250	59.00	59.02	59.09
20	6	62.5	1	250	58.92	59.02	59.09
21	6	62.5	1	250	58.96	59.02	59.09

The experimental range and levels of the independent variables used are presented in Table 1.The experimental data obtained were subjected to the second-order polynomial regression model. The response, *Y* can be related to the independent variables as a polynomial model based on the following quadratic equation [[35], [36], [37]]:

(4) $$Y=\;b_{0}+\;b_{1}A+\;b_{2}B+\;b_{3}C+\;b_{4}D+\;b_{11}A^{2}+\;b_{22}B^{2}+\;b_{33}C^{2}+\;b_{44}D^{2}+\;b_{12}AB+\;b_{13}AC+\;b_{14}AD+\;b_{23}BC+\;b_{24}BD+b_{34}CD$$

Where *Y* is the predicted output response (removal efficiency); *A* is the initial pH, *B* is the contact time (min), *C* is the Ho-CaWO_4_NPs dose (g/L) and *D* is the initial MB concentration (mg/L); ($b_{0}$, $b_{1}$, $b_{2}$, $b_{3}$ and ${\;b}_{4}$), ($b_{11}$, $b_{22}$, $b_{33}$ and $b_{44}$), and ($b_{12}$, $b_{13}$, $b_{14}$, $b_{23}$, $b_{24}$ and $b_{34}$) are the constant regression coefficients for the linear, quadratic and interaction effects, respectively.

The analysis of variance (ANOVA) was employed to evaluate the adequacy of the developed model and the statistical significance of the constant regression coefficients. ANOVA was also used to examine the individual, the interactive and the quadratic effects of the process variables on the removal efficiency of MB using Ho-CaWO_4_ nanoparticles. The model terms were assessed using the p-value with a confidence level of 95%. The Fisher’s F-value was used to examine the significance of the regression coefficients. Also, the coefficient of determination (R^2^) value was compared to the adjusted R^2^ value to check the adequacy of the model. Three-dimensional (3D) surface and two-dimensional (2D) contour plots of the independent variables’ interactive effects with their corresponding responses were made using the Design expert (8.0.7.1 trial version) to observe the interaction between the process variables with their corresponding effect on the output response. Finally, the optimum values of the independent variables were determined using the same software. The Artificial Neural Network (ANN) was used also to predict the output responses using the MATLAB software [39] which was compared to the responses generated by the CCD with the actual experimental values. The root mean squared error (RMSE) and the absolute average deviation (AAD) were applied to determine their performances and capabilities in predicting the responses.

## Results and discussion

### Characterization of the synthesized Ho-CaWO_4_ nanoparticles

**Fig. 1:**
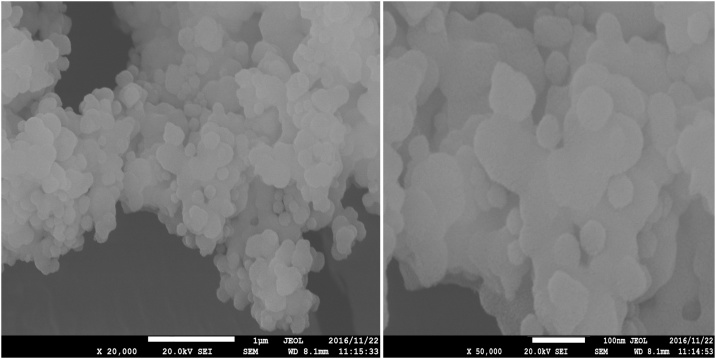
FE-SEM images of Ho-CaWO_4_ nanoparticles.

**Fig. 2:**
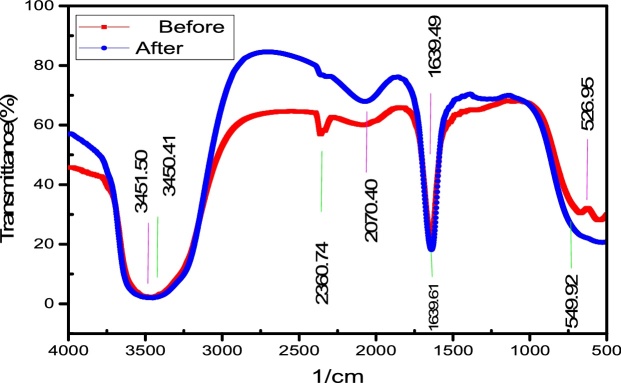
FTIR spectra of Ho-CaWO_4_ nanoparticles before and after MB adsorption.

The surface electron microscopy (SEM) images (20 and 50 kX) of the adsorbent used in this study, Ho-CaWO_4_ nanoparticles is shown Fig. 1. The images reveal that the Ho-CaWO_4_ nanoparticle is in nanoscale. Fourier Transform Infrared Spectroscopy (FT-IR) was used to characterize the functional groups present in the Ho-CaWO_4_ nanoparticles before and after adsorption of MB. The FTIR of Ho-CaWO_4_ nanoparticles before and after adsorption is shown in Fig. 2 which was recorded in the range of 400–4000 cm^−1^. The FT-IR analysis on the Ho-CaWO_4_ NPs before MB adsorption shows the presence of C–Br stretching of alkyl halides (549.92 cm^−1^), N—H bending of 1° amines (1639.61 cm^−1^), C≡N stretching of nitriles (2360.79 cm^−1^), —C≡C— stretching of alkynes (2070.52 cm^−1^) and O—H stretching, H—bonded of alcohols and phenols (3450.41 cm^−1^). O—H stretch, H—bonded of alcohols and phenols is a very broad and strong band which took an active part in the adsorption of MB because of the presence of hydrogen bonding [38]. After the adsorption of MB, the intensities of the bands were shifted from 549.92, 1639.61, 2360.79, 2070.52 and 3450.41to526.95, 1639.49, 2070.40, 2360.71and 3451.50 cm^−1^. This shift in the peaks indicates the binding of dye ions on the adsorbent.

### RSM modelling

The adsorption experiments were performed according to Table 2. The generated data were analyzed using the Design expert version 8.0.7.1 software, USA and then interpreted. The actual response values were close to the predicted values for a specific experimental run (Fig. 4 and Table 2).

**Fig. 3:**
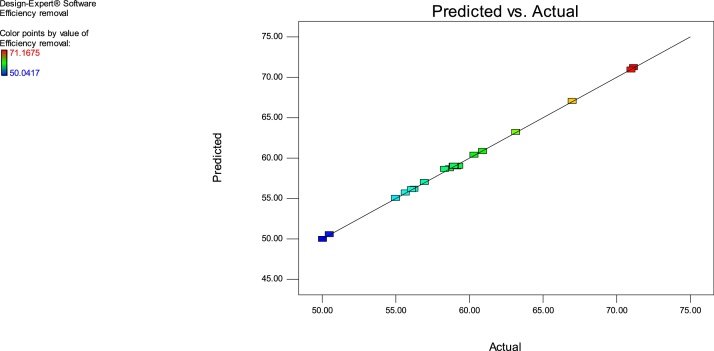
The predicted values versus the observed values of MB adsorption on Ho-CaWO_4_ nanoparticles.

**Fig. 4:**
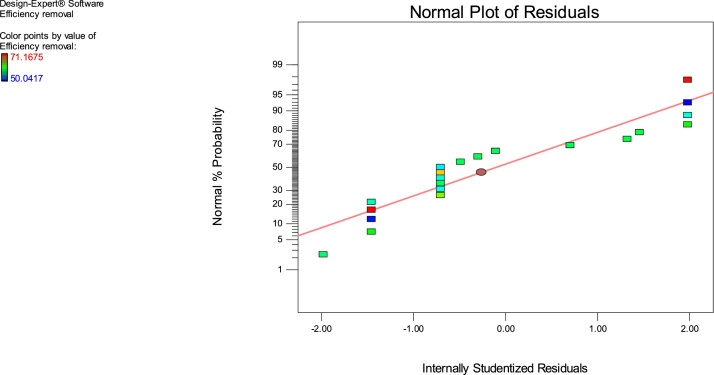
The normal %probability residuals and studentized residuals for MB adsorption onto Ho-CaWO_4_ nanoparticles.

### Model fitting and ANOVA analysis

Table 3 presents the ANOVA results for the developed response surface quadratic model obtained. The ANOVA indicates whether the response surface quadratic model developed is statistically suitable for the representation of the process of MB adsorption on Ho-CaWO_4_ nanoparticles at the studied range. The model Fisher’s F-value of 835.76 implies the model is significant. There is only a 0.01% chance that an F-value of a model this large could occur due to noise. P-values less than 0.05 indicate the model terms that are significant [35]. In this case, A, B, D, BD, CD, A^2^, B^2^ and D^2^ are the significant model terms. P-values greater than 0.10 indicate the model terms that are not significant. The p-value is the probability of rejecting a null hypothesis. The higher the Fisher’s F-value, the more significant the individual coefficients and the more adequate the model [39].

**Table 3:** ANOVA for the response surface quadratic model.

Source	Sum of		Mean	F	p-value	
	Squares	df	Square	Value	Prob > F	
Model	576.98	14	41.21	835.76	< 0.0001	significant
A-pH	28.12	1	28.12	570.23	< 0.0001	
B-Time	3.33	1	3.33	67.62	0.0002	
C-Nano	0.25	1	0.25	5.12	0.0644	
D-Concentration	72.00	1	72.00	1460.10	< 0.0001	
AB	0.069	1	0.069	1.40	0.2817	
AC	0.13	1	0.13	2.54	0.1622	
AD	0.11	1	0.11	2.20	0.1887	
BC	0.032	1	0.032	0.64	0.4527	
BD	0.32	1	0.32	6.54	0.0431	
CD	0.31	1	0.31	6.37	0.0450	
A^2^	0.48	1	0.48	9.79	0.0203	
B^2^	6.59	1	6.59	133.64	< 0.0001	
C^2^	0.14	1	0.14	2.78	0.1465	
D^2^	10.39	1	10.39	210.79	< 0.0001	
Residual	0.30	6	0.049			
Lack of Fit	0.19	2	0.097	3.78	0.1198	not significant
Pure Error	0.10	4	0.026			
Cor Total	577.28	20				

R^2^ = 0.9995, Adj. R^2^ = 0.9983, Pred. R^2^ = 0.9233, Adequate precision = 113.257.

The lack of fit F-value of 3.78 entails the lack of fit is not significant relative to the pure error. There is an 11.98% chance that a lack of fit F-value this large possibly will occur due to noise. Non-significant lack of fit is good. Also, the p-value of lack of fit is greater than 0.05; this implies that the model fits the experimental data and the independent process variables have a significant effect on the response. The coefficients of a particular process variable and two combined variables explain the extent of the effect of that variable and the interaction between two variables, respectively [35]. The effect of the terms on the model using the F-value is in this order: D > A > D^2^ > B^2^ > B > A^2^ > BD > CD > C > C^2^ > AC > AD > AB > BC. The initial MB concentration was found to have the greatest influence on the model followed by the pH of the solution. The predicted R^2^ (0.9233) is in reasonable agreement with the adjusted R^2^ (0.9983). The coefficient of determination, R^2^ of 0.9596 which is the degree of fitness confirms the high correlation between the predicted and the experimental responses. These values are close to unity which confirms the validity of the model [40]. The signal to noise ratio is measured by the adequate precision; a ratio greater than 4 is desirable. The adequate precision ratio of 113.257 indicates an adequate signal. This model can be used to navigate the design space.

The quadratic model equation relating the response (MB removal efficiency) and the independent process variables (initial pH, contact time, Ho-CaWO_4_ nanoparticles dose and initial MB concentration) is given by Eq. (5):

(5) Y = 59.02 − 3.75A − 1.29B + 0.16C − 6.00D + 0.21AB − 0.13AC − 0.26AD − 0.063BC + 0.4BD + 0.20CD + 0.43A2 − 1.61B2 − 0.23C2 + 2.02D2

The insignificant terms of the above model equation can be removed for the accurate prediction of the output response [41,42]. As seen in the normal probability plots of the residuals (normal % propability versus internally studentized residuals) (Fig. 4), great deviation from ordinariness was not seen. The graphical points follow a straight line and therefore, no transformation of data is required [43].

### Response surface plots

**Fig. 5:**
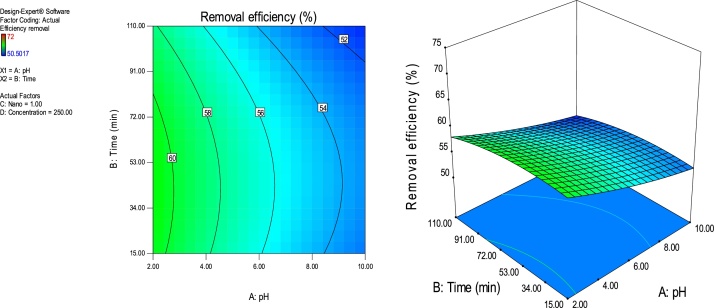
2D contour and 3D surface plots of the effect of pH and time on MB removal efficiency using Ho-CaWO_4_ nanoparticles at constant nano dosage and concentration.

**Fig. 6:**
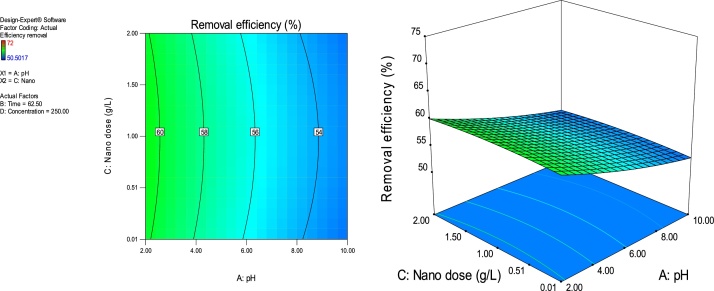
2D contour and 3D surface plots of the effect of pH and nano dose on MB removal efficiency using Ho-CaWO_4_ nanoparticles at constant time and concentration.

**Fig. 7:**
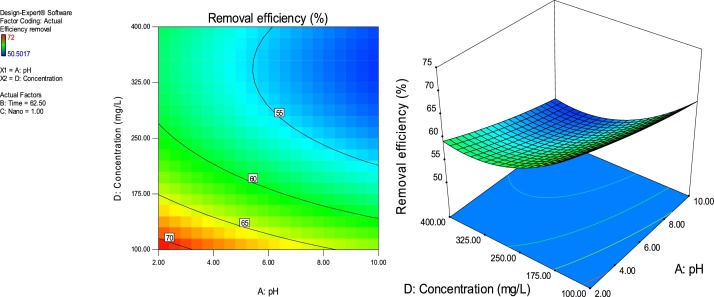
2D contour and 3D surface plots of the effect of pH and concentration on MB removal efficiency using Ho-CaWO_4_ nanoparticles at constant time and nano dose.

**Fig. 8:**
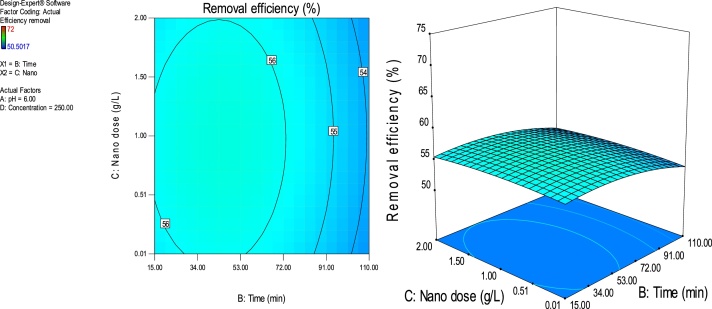
2D contour and 3D surface plots of the effect of time and nano dose on MB removal efficiency using Ho-CaWO_4_ nanoparticles at constant pH and concentration.

**Fig. 9:**
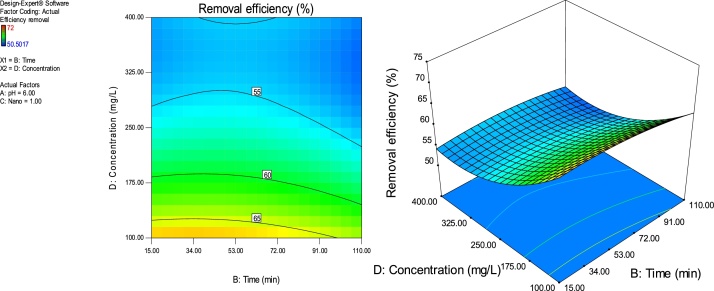
2D contour and 3D surface plots of the effect of time and concentration on MB removal efficiency using Ho-CaWO_4_ nanoparticles at constant pH and nano dose.

**Fig. 10:**
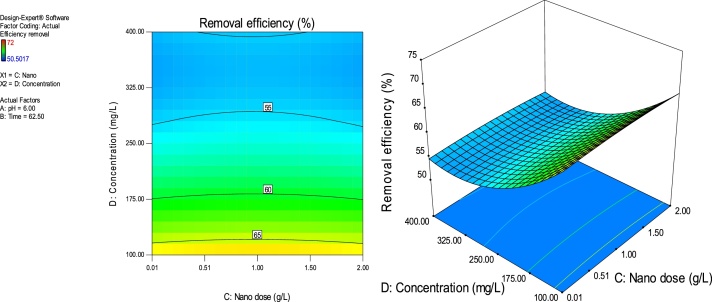
2D contour and 3D surface plots of the effect of nano dose and concentration on MB removal efficiency using Ho-CaWO_4_ nanoparticles at constant time and pH.

RSM is a statistical technique for the study of the combined effects of independent process variables on a response or responses [41].To study the interaction of the different process variables and their corresponding effects on the response (MB removal efficiency),two-dimensional (2D) contour plots and three-dimensional (3D) response surface plots against any two independent process variables were made while keeping the other process variables at their central (0) level. Fig. 5, Fig. 6, Fig. 7, Fig. 8, Fig. 9, Fig. 10 presents the 2D contour and 3D surface plots made for the interactions between the process variables with their respective output responses. Adsorption processes are significantly influenced by the pH of the solution which is also related to the functional groups present on the adsorbing material and the chemistry of solution [44,45]. Fig. 5, Fig. 6, Fig. 7 show that the adsorption of MB on Ho-CaWO_4_ NPs was decreased with increasing pH. Fig. 5 shows that maximum removal of 70% was achieved at a pH of 2.6 and time of 80 min. The adsorption process was more favorable in the acidic range because of the electrostatic attractions between the positively charged surface of the Ho-CaWO_4_ nanoparticles and the anionic dye (MB). Fig. 7 shows that optimum removal of 70.1% was achieved at pH of 2.4 and concentration of 115 mg/L.65% removal was achieved at a concentration of 125 mg/l and time of 15 min (Fig. 9). Time of contact is a very important parameter in all processes. The adsorption of the adsorbate, MB was improved with increasing time of contact and dosage of Ho-CaWO_4_ nanoparticles relatively (Fig. 8). The increase in MB removal efficiency with Ho-CaWO_4_ nanoparticles dose and time is due to the availability of more active adsorption sites for the trapping of the dye and presence of enough time for the adsorption process, respectively [44]. A negative effect on the adsorption process can be viewed at the interaction between concentration and pH (Fig. 7) and concentration and Ho-CaWO_4_ nanoparticles dose (Fig. 10). The adsorption of MB on Ho-CaWO_4_ nanoparticles was found to decrease with increasing concentration owing to the adsorbent surface is saturated with the adsorbate [46].

### Artificial Neural Network (ANN) modelling

Artificial Neural Networks (ANN's) is used for predicting the outcome and behavior of systems, designing different processes, and analyzing already existing processes [22]. The Multi-layer perceptron (MLP) is usually trained with back-propagation (BP) algorithm. In the MLP networks, error minimization can be achieved by using gradient descent (GD), conjugate gradient (CG) and Levenberge–Marquardt (LM) methods [28]. The input and output for training were obtained from the experiments planned through the CCD. The multilayer perceptron (MLP) technique used in this work was developed in MATLAB (The Math Works Inc. 2018a) with four input neurons which are the independent variables (initial pH, contact time, Ho-CaWO_4_ nanoparticles dose, and initial MB concentration), a hidden layer of eight neurons and an output layer of one neuron representing the removal efficiency of MB on Ho-CaWO_4_ nanoparticles.

The Neural Fitting app (nftool) was used to select data, create and train a network. Its performance was evaluated using the mean square error (MSE) and regression analysis coefficient (R^2^) present in the MATLAB software.

A two-layer feed-forward network with sigmoid hidden neurons and linear output neurons (fitnet) can fit multi-dimensional mapping problems arbitrarily well given consistent data and enough neurons in its hidden layer. The network MLP (4:8:1) was trained with the Leven berg-Marquardt backpropagation algorithm (trainlm). This algorithm normally needs more memory but less time. Training automatically discontinues when generalization ceases to improve, as indicated by an increase in the mean square error (MSE) of the validation samples.

**Fig. 11:**
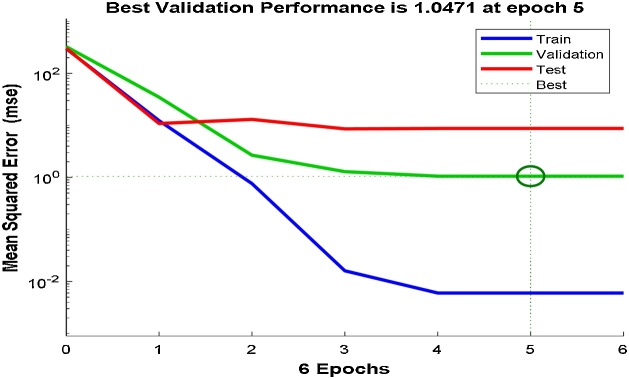
Performance plot for the ANN model.

**Fig. 12:**
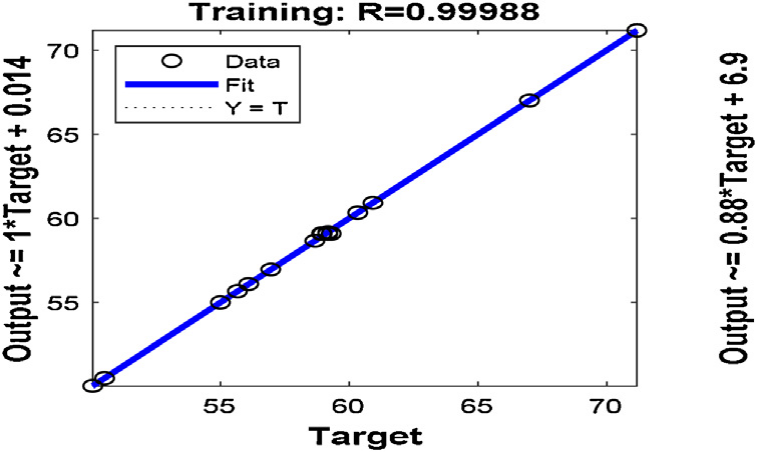
The actual values versus the predicted values using the ANN.

To getter a better prediction of the output response, the best number of neurons in the hidden layer, training samples, validating samples and testing samples were chosen by the trial-and-error method. A total of 21 samples were used for the ANN modeling; 75% (16 samples), 15% (3 samples) and 10% (2 samples) were used to training, validation of the training and testing, respectively. After the selection of the best number of neurons for the hidden layer by trial-and-error, the network was trained for 6 iterations. The MSE of the trained network is 6.01718e-3 with regression coefficient, R^2^ of 0.999881. The regression coefficient measures the correlation between the predicted responses (outputs) and the experimental responses (targets). An R-value close to 1 implies a better relationship. Fig. 11, Fig. 12 show the performance plots of the trained network and the regression plots, respectively. Table 2 also shows the predicted responses using the ANN modeling technique.

**Fig. 13:**
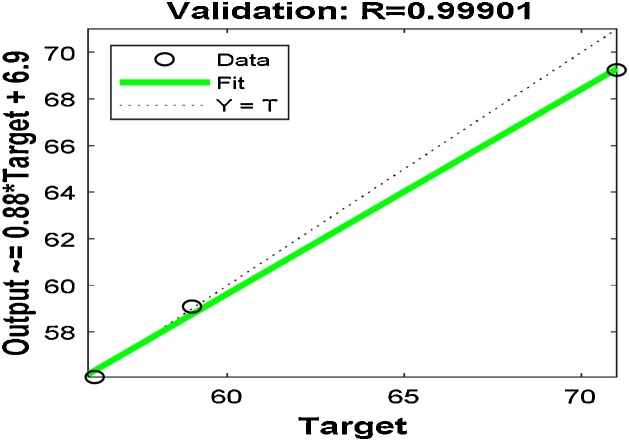
Validation regression plot.

The linear fit model obtained by the plot of the ANN validation outputs, *Y* versus the targets, *T* (the experimental value) is shown in Fig. 13 and Eq. (7)

(7) *Y* = (0.88) *T* + (6.9)

This model was used to predict the ANN model output response values.

### Comparison of RSM and ANN

The root mean squared error (RMSE) and the absolute average deviation (AAD) were used to establish the performance and the best modelling technique to predict the output responses. The RMSE and ADD were evaluated as follows [22]:

(6) RMSE=(1n∑i=1n(%Ri,pred- %Ri,exp)2)12

(7) $$AAD=[\frac{1}{n}\sum_{i=1}^{n}(\frac{{\text{\%}R}_{i,pred}-\;{\text{\%}R}_{i,exp}}{{\text{\%}Ri,}_{exp}})]\;X\;100$$

Where *n* is the number of data points or samples, ${\%R}_{i,pred}$ is the predicted value and ${\%R}_{i,exp}$ is the experimental value.

The AAD for RSM and ANN were determined as 0.001 and 0.320 while the RMSE for RSM and ANN were obtained as 0.119 and 0.993, respectively. The minimum RMSE and AAD are the best. The RSM model is more acceptable since it has a lower RMSE and AAD values compared to that of ANN. This may be owing to the limited number of experimental runs used in the present study. Generally, the ANN requires a very large number of data points to perform better in the training of networks [22,47]. From Fig. 3, Fig. 12, it is apparent that both models (RSM and ANN) could capably predict the removal of MB onto Ho-CaWO_4_ NPs. Therefore, RSM was used further for the optimization of the MB adsorption on Ho-CaWO_4_ nanoparticles.

### Numerical optimization using CCD-RSM

**Fig. 14:**
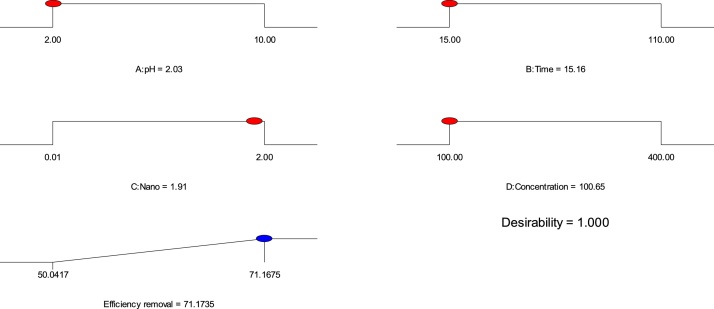
The desirability effect for MB adsorption on Ho-CaWO_4_ nanoparticles.

Optimization was successfully done using the Design expert software (Stat-Ease, 8.0.7.1 trial version) to define the optimum conditions for MB adsorption on Ho-CaWO_4_NPs. The optimum predicted conditions for maximum MB removal and the optimum removal efficiency are presented in Table 4. The experimental value of 70.96% obtained by performing an experiment at the optimum parametric conditions stated in Table 4; this was found to be close to the predicted MB removal efficiency of 71.17%. Roslan et al. [48] stated that a generated model is acceptable if the desirability value is close to unity. A desirability of 1.000 confirms the acceptance and applicability of the model (Table 4 and Fig. 14).

**Table 4:** The optimum predicted conditions for maximum MB removal on Ho-CaWO_4_ nanoparticles.

pH	Time (min)	Ho-CaWO_4_ NPs dose (g/L)	Concentration (mg/L)	Efficiency removal (%)	Desirability
2.03	15.16	1.91	100.65	71.17	1.000

### Adsorption isotherms

The equilibrium adsorption isotherm is important in the design of adsorption systems. Adsorption isotherms are applied to determine the relationship between the amount of adsorbate and its equilibrium concentration in solution [49]. There are several isotherm equations but the three most commonly used isotherms (Langmuir, Temkin and Freundlich) were used in this study. The adsorption isotherm experiment was performed at pH of 4 and temperature of 298 K for 90 min using Ho-CaWO4 nanoparticles dose of 0.05 g/L.

The Langmuir isotherm model is presented in Eq. (3) [50]:

(8) $$\frac{C_{e}}{q_{e}}=\frac{1}{q_{m}}.\frac{1}{k_{L}}+\frac{C_{e}}{q_{m}}$$

Where *q*_e_ is the metal uptake (mg/g) by Ho-CaWO_4_ nanoparticles (mg/g), *q*_m_ is the maximum/monolayer adsorption capacity (mg/g), *K*_L_ is the Langmuir isotherm constant related to the affinity of the binding sites and energy of adsorption (L/mg). The separation factor or equilibrium parameter, *R_L_* is defined as [50]:

(9) $$R_{L}=\frac{1}{1+K_{L}C_{0}}$$

The *R_L_* value indicates whether the isotherm is either favorable (0 < *R_L_* < 1), unfavorable (*R_L_* > 1), linear (*R_L_* = 1) or irreversible (*R_L_* = 0) [36,51].

The Freundlich isotherm is shown in Eq. (4) [52]:

(10) $$Logq_{e}=\frac{1}{n}\log C_{e}+\log k_{f}$$

Where *q*_e_ is the amount of MB adsorbed (mg/g), *C*_e_ is the equilibrium concentration of MB in solution (mg/L), and *K*_f_ and *n* are the constants incorporating the factors affecting the adsorption capacity and intensity of adsorption, respectively.

The Temkin isotherm can be expressed as [14]:

(11) $$q_{e}=\;B_{1}Ln\left(A_{T}\right)+\;B_{1}Ln(C_{e})$$

A plot of *q_e_* versus *Ln C_e_* enables the determination of the constants, *A_T_* and *B*_1_. *B*_1_ is the heat of sorption and *A_T_* is the equilibrium binding constant; where *B*_1_ = *RT*/*b*, *T* is the absolute temperature (K) and *R* is the universal gas constant (8.314 J mol^−1^ K^−1^).

**Fig. 15:**
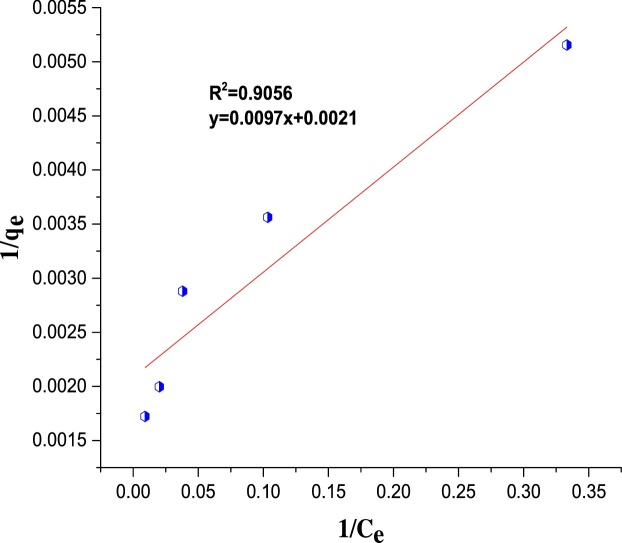
The plot of Langmuir isotherm for MB dye adsorption on Ho-CaWO_4_ nanoparticles.

**Fig. 16:**
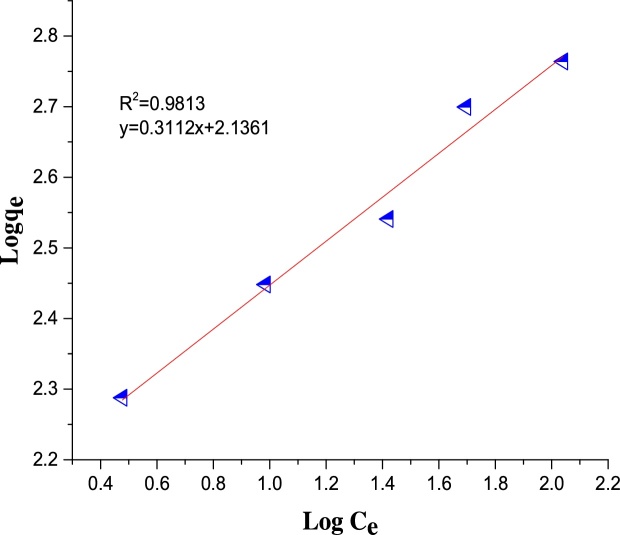
The plot of Freundlich isotherm for MB dye adsorption on Ho-CaWO_4_ nanoparticles.

**Fig. 17:**
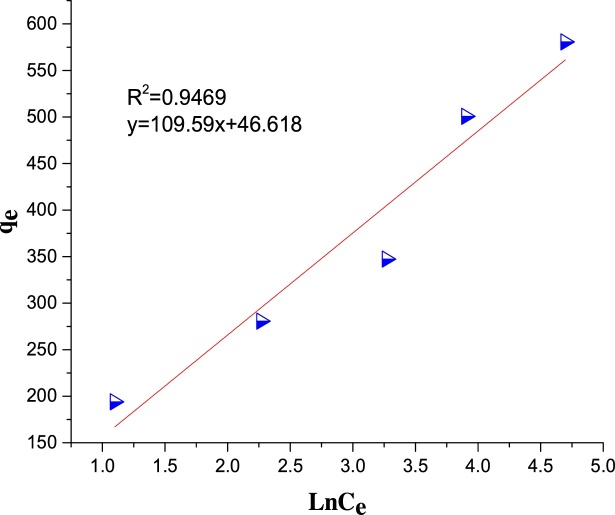
The plot of Temkin isotherm for MB dye adsorption on Ho-CaWO_4_ nanoparticles.

The regression coefficient, R^2^ was used as the basis for choosing the best appropriate isotherm for the adsorption process. The values of the calculated isotherm parameters along with the regression coefficients are listed in Table 5. The isotherm data was found to be more compatible with the Freundlich isotherm with R^2^ of 0.9813 which is higher than R^2^ of the other adsorption isotherms (Table 5 and Fig. 15, Fig. 16, Fig. 17).The *R_L_* value of 0.002 indicates that the adsorption of MB on Ho-CaWO_4_ nanoparticles is favorable since 0 < 0.002 < 1. Moreover, the intensity of adsorption, 1/*n* was found to be 0.3112. This value is less than one, it indicates the adsorption of MB on Ho-CaWO_4_ nanoparticles is favorable [53]. The monolayer adsorption capacity was found to be 103.09 mg/g.

**Table 5:** Isotherms parameters for adsorption of MB onto Ho-CaWO_4_ nanoparticles at temperature of 298 K.

Langmuir		Temkin		Freundlich	
*q_m_*(mg/g)	103.09	*B_T_*(kJ/mol)	109.59	*n*	3.214
*k_L_*(L/mg)	4.63	*A_T_*(L/g)	1.54	1/*n*	0.3112
*R_L_*	0.002	*b_T_* (kJ/mol)	0.023	*k_f_*	136.8
R^2^	0.9056	R^2^	0.9469	R^2^	0.9813

### Adsorption kinetics

The MB adsorption kinetic data were fitted into the pseudo-second-order and pseudo-first-order models. The mechanism of the adsorption process was also determined. The intraparticle diffusion plot is usually used to identify the mechanism involved in adsorption processes [54]. The Lagergren (pseudo-first-order) rate equation is defined as Eq. (12) [55]:

(12) $$Log(q_{e}-q_{t})=Log(q_{e})-\frac{k_{1}}{2.303}t$$

Where *q_t_* and *q_e_* are the amounts adsorbed at time *t* and at equilibrium (mg/g) and *k_1_* is the pseudo-first-order rate constant for the adsorption process (min^−1^).

The Ho (pseudo-second-order) kinetic model can be represented in the following form [56]:

(13) $$\frac{t}{q_{t}}=\;\frac{1}{K_{2}{q_{e}}^{2}}+\;\frac{t}{q_{e}}$$

where *K_2_* is the pseudo-second-order rate constant (g mg^−1^ min^−1^); *q_e_* and *q_t_* are the amounts of adsorbate adsorbed on the adsorbent (mg/g) at equilibrium and at time *t*.

Adsorption is a thermodynamic system in which different compounds are in competition to reach an equilibrium state. In an adsorption phenomenon, the adsorbing molecules should be transferred from the solution mass phase to the level of the solvent film surrounded the absorbent particle. This phase is called the film diffusion process. The MB adsorption on Ho-CaWO_4_ nanoparticles may be controlled by film or intraparticle diffusion. The intraparticle diffusion equation is expressed as [[56], [57], [58]]:

(14) $$q_{t}=K_{p}t^{0.5}+c$$

Where c is a constant that provides an idea of the thickness of the boundary layer and *K_p_* is the intraparticle diffusion rate constant (mg/g min^1/2^); *q_t_* is the amount of MB adsorbed (mg/g) at time *t* (min).

**Fig. 18:**
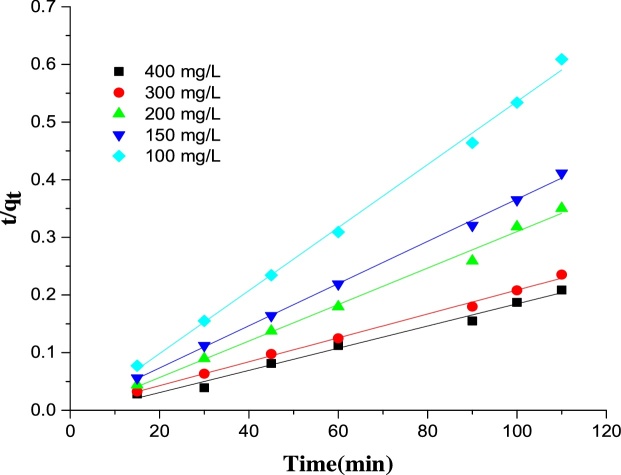
The plot of pseudo-second-order kinetic for MB dye adsorption on Ho-CaWO_4_ nanoparticles (adsorbent dose: 0.05 g/L,pH:4, temperature: 298 K).

The correlation coefficient, R^2^ values for the pseudo-second-order (Ho) model (Table 6 and Fig. 18) was higher than that of the pseudo-first-order model. This suggests that the adsorption of MB on Ho-CaWO_4_ nanoparticles is chemisorption in nature [54]. The values of *c* from the intraparticle diffusion equation were not close to the origin indicating the insignificance of the liquid film diffusion in rate determination of the adsorption process [59]. The R^2^ value for intraparticle diffusion model was not high, thus also showing the irrelevance of the film diffusion as a rate determining factor in the process [59].

**Table 6:** Kinetics parameters estimated for the adsorption of MB onto Ho-CaWO_4_ nanoparticles.

*C*_0_	Lagergren			Ho			Intraparticle diffusion		
	*q_e_*	*K*_1_	R^2^	*q_e_*	*K*_2_	R^2^	*K_p_*	*c*	R^2^
100	37.55	0.0038	0.4913	181.8	0.0027	0.9969	1.6387	178.2	0.645
150	88.02	0.00046	0.1463	270.3	0.142	0.9985	0.414	275.3	0.414
200	182.01	0.0011	0.306	312.5	0.002	0.9933	3.095	306.4	0.366
300	235.23	0.0007	0.241	476.2	0.0034	0.9967	2.073	490.9	0.133
400	338.76	0.0025	0.1517	526.3	0.0005	0.99889	15.117	459.8	0.188

## Conclusion

The applicability of Ho-CaWO_4_ nanoparticles for the removal of Methylene Blue (MB) from aqueous solution using the adsorption process was studied. The Ho-CaWO_4_NPs was prepared using the hydrothermal method of synthesis. The effects of different process variables such as pH, contact time, Ho-CaWO_4_ nanoparticles dose and initial MB concentration on the removal of MB using Ho-CaWO_4_ nanoparticles were investigated using the central composite design (CCD) method. The capabilities of the Response Surface Methodology (RSM) and Artificial Neural Network (ANN) modeling methods in predicting the output response (MB removal efficiency) were examined. The interactive effects of the process variables and their optimum conditions were determined. The adsorption data were fitted into different isotherm and kinetics models. The RSM model found to be more acceptable since it has a lower RMSE and AAD compared to the ANN values but both can be applied for the prediction of the output (MB removal efficiency). Optimum MB removal of 71.17% was obtained at pH of 2.03, contact time of 15.16 min, Ho-CaWO_4_ nanoparticles dose of 1.91 g/L, and MB concentration of 100.65 mg/L. The experimental followed the Freundlich isotherm and pseudo-second-order kinetic model than the other models. Maximum adsorption capacity of 103.09 mg/g was obtained. From the present study, it can be concluded that the prepared Ho-CaWO_4_ nanoparticles can be used for the removal of MB from its aqueous solutions and the process can also be optimized.

## Funding sources

This work was supported by the Research Grant of the Environmental Health Laboratory of Zabol Province, Iran (Grant No. IR.ZBMU.REC-1397-028).

## Declaration of Competing Interest

Authors have no conflict of interests.
